# In Vivo Repeatedly Charging Near‐Infrared‐Emitting Mesoporous SiO_2_/ZnGa_2_O_4_:Cr^3+^ Persistent Luminescence Nanocomposites

**DOI:** 10.1002/advs.201500001

**Published:** 2015-02-09

**Authors:** Zhanjun Li, Yuanwei Zhang, Xiang Wu, Xiaoqiong Wu, Rohit Maudgal, Hongwu Zhang, Gang Han

**Affiliations:** ^1^ Department of Biology and Molecular PharmacologyUniversity of Massachusetts Medical SchoolWorcesterMA01605USA; ^2^ Institute of Urban EnvironmentChinese Academy of SciencesJimei Road 1799Xiamen361021China

**Keywords:** imaging, luminescence, mesoporous, near‐infrared, persistent

## Abstract

Near‐infrared (NIR) persistent phosphor ZnGa_2_O_4_:Cr^3+^ (ZGC) has unique deep‐tissue rechargeable afterglow properties. However, the current synthesis leads to agglomerated products with irregular morphologies and wide size distributions. Herein, we report on in vivo rechargeable mesoporous SiO_2_/ZnGa_2_O_4_:Cr^3+^ (mZGC) afterglow NIR‐emitting nanocomposites that are made by a simple, one‐step mesoporous template method. At less than 600 °C, pores in mesoporous silica nanoparticles (MSNs) act as nanoreactors to generate in situ ZnGa_2_O_4_:Cr^3+^ NIR‐persistent phosphors. The as‐synthesized mZGC preserves defined size, morphology, and mesoporous nanostructure of the MSNs. The persistent luminescence of the as‐synthesized mZGC is recharged in a simulated deep‐tissue environment (e.g., ≈8 mm pork slab) in vitro by using red light (620 nm). Moreover, mZGC can be repeatedly activated in vivo for persistent luminescence imaging in a live mouse model by using white LED as a light source. Our concept of utilizing mesoporous silica as nanoreactor to fabricate ZGC PL nanoparticles with controllable morphology and preserved porous nanostructure paves a new way to the development and the wide application of deep tissue rechargeable ZGC in photonics and biophotonics.

## 1 Introduction

Near‐infrared (NIR) persistent luminescence (PL) is an intriguing phenomenon in which PL phosphors can store excitation energy in energy traps while continuing to emit photons for weeks after excitation ceases.^1^, ^2^, ^3^ The temporal separation of excitation and afterglow properties of these persistent phosphors makes them ideal as in vivo optical imaging contrast reagents.^4^, ^5^, ^6^ Until now, persistent luminescence has relied on short‐wavelength excitation (e.g., ultraviolet light) which has rather limited tissue‐penetration depth.^7^, ^8^, ^9^, ^10^ To address this problem, a NIR‐light‐stimulated PL mechanism was proposed in LiGa_5_O_8_:Cr^3+^, to release energy trapped in deeper energy levels of the phosphor, but in this case, the energy must be precharged by UV‐light and the photostimulated emission continues to weaken after each cycle of photostimulation and will finally become extinguished.^3^, ^11^ Very recently, the PL phosphor, ZnGa_2_O_4_:Cr^3+^ (ZGC), was found to be activatable by using tissue‐penetrable red light, which means that energy can be recharged and NIR PL imaging is no longer limited by the luminescence‐decay life‐time of the phosphor.^12^ Thus ZGC is arguably the optimal rechargeable NIR persistent emitting phosphor reported to date. Despite such inspiring progress, the production of uniformly structured NIR PL ZGC phosphors remains challenging. To make such NIR‐persistent phosphors bulk crystal requires temperatures >750 °C in traditional solid‐state annealing reactions.^1^, ^7^, ^13^ Moreover, to convert such bulk crystal into nanoparticles that are sufficiently disperse for biological applications, certain tedious physical treatments such as grinding^2^, ^3^, ^14^, ^15^ or laser ablation^16^ must be utilized. The afforded products are generally highly heterogeneous and suffer from severe agglomeration.

Because of their readily controllable synthesis and resulting morphology, super‐high specific surface area, huge pore volume, and good biocompatibility, mesoporous silica nanoparticles (MSNs) are widely utilized in biology, drug delivery, and medicinal applications to encase various functional molecules/luminescence contrast reagents.^17^, ^18^, ^19^ We propose that MSNs may be used as a template to synthesize ZGC NIR persistent phosphors in situ with defined size and morphology (mZGC). Since temperatures beyond 750 °C may lead to the collapse of the mesoporous silica nanostructures, the reaction temperature for the phosphor synthesis was explored systematically in this study. We found that at 600 °C, the as‐synthesized mZGC preserves defined size, morphology and mesoporous nanostructure of the MSNs as well as possess the optimal luminescence property. Further, the performance of mZGC in imaging was measured both in vitro and in vivo to assess potential applications in biophotonics. We demonstrated that as‐synthesized mZGC was able to be recharged in a simulated deep‐tissue environment (≈8 mm pork slab) in vitro by using red light. Moreover, we observed that mZGC was able to be repeatedly activated in vivo for persistent luminescence imaging in a live mouse model by using white LED as a light source.

## 2 Results and Discussion

### 2.1 Formation of mZGC by Using MSNs as Nanoreactors Morphology and Porous Structure of mZGC

**Scheme 1:**
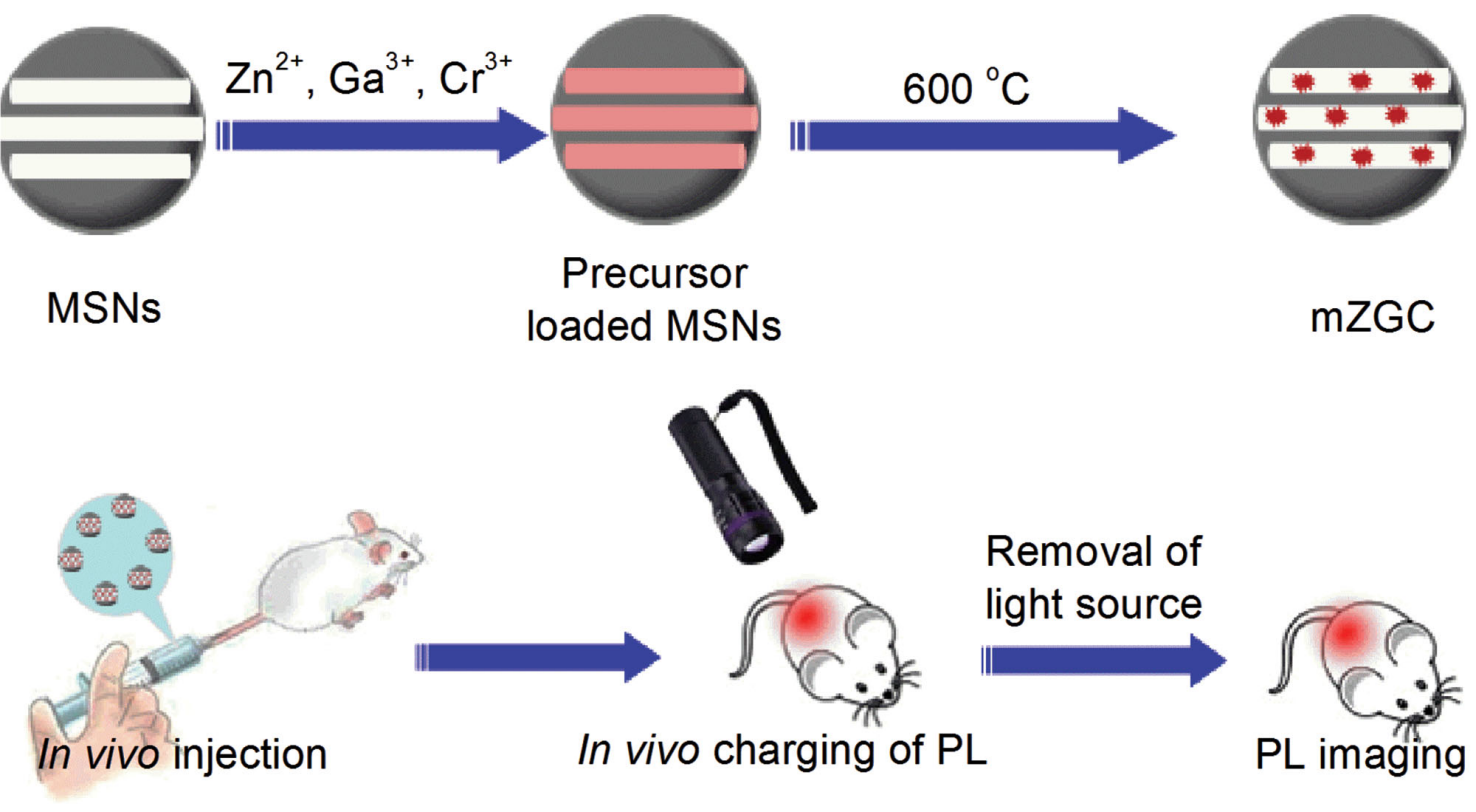
Illustration of the synthesis of PL‐functionalized MSNs and their in vivo imaging application.

The MSNs were impregnated with ZGC nitrate precursor solutions which entered the nanochannels of MSNs with ease due to the capillarity of the mesopores. The optimal used composition was determined at Zn/Ga/Cr = 1/1.997/0.003 by molar ratio (Figure S1, Supporting Information). ZGC was formed in the nanochannels of the MSNs after vacuum drying and annealing, as shown in **Scheme** **1**. By measuring the mass of the used silica and the total mass of the as‐formed nanocomposites, the content of ZGC in mZGC is calculated to be 10.4% by weight (Table S1, Supporting Information). Such NIR‐persistent‐luminescent mesoporous nanocomposites, which combine the unique optical properties of NIR persistent phosphors and the mesoporous attributes of mesoporous silica, can be produced by our simple, one‐step mesoporous template method.

**Figure 1:**
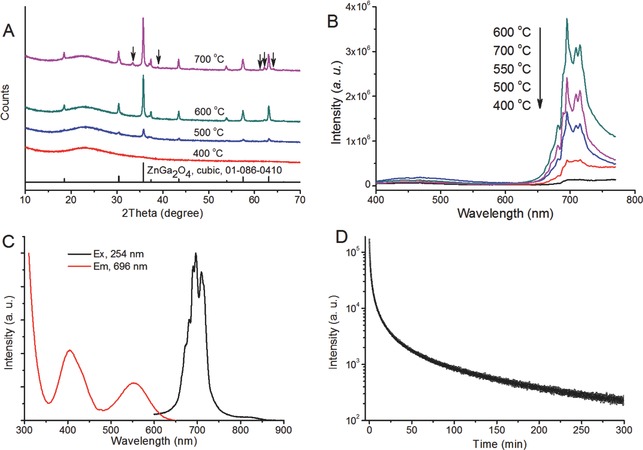
Optimization of the experimental conditions. A) XRD and B) photoluminescence spectra (excitation wavelength, 254 nm) of the mZGC samples synthesized at various temperatures, C) PL excitation and emission spectra (tested by using the phosphorescence mode of the fluorimeter), D) PL decay curve of mZGC synthesized at 600 °C, excited by UV lamp (254 nm). Sample mass for above measurements are 100 mg.

X‐ray diffraction (XRD) and luminescence measurements were used to confirm the nature of the as‐synthesized nanocomposite. The XRD results (**Figure** **1**A) show that the diffraction characteristics (peaks at 30.4°, 35.78°, 43.50°, 57.48°, 63.12°) of the ZGC crystal already appear when the annealing temperature reaches 500 °C; they then become clear when the temperature increases to 600 °C, which is in agreement with the presence of the spinel phase ZnGa_2_O_4_ (JCPDS index no. 01–086–0410). However, impurity peaks start to appear at 700 °C, which indicate the formation of metal silicates (arrows in Figure 1A). As a result, we observed that the PL intensity (Figure 1B; 650–750 nm) initially increases with temperature (from 400–600 °C) and reaches the optimal intensity at 600 °C before decreasing at 700 °C.

The phosphorescence excitation spectrum of the sample synthesized at 600 °C consists of three ZGC characteristic excitation bands (Figure 1C). These bands, which range from <350, 350–470, and 470–650 nm, can be attributed to the band‐to‐band transitions of ZnGa_2_O_4_, ^4^A_2_→^4^T_1_, and the ^4^A_2_→^4^T_2_ transition of Cr^3+^, respectively.^20^ Among these bands, the absorbance at 600–650 nm is responsible for the in vivo recharging by using deeper‐tissue‐penetrating red light.^12^ The PL spectrum (Figure 1C) of the mZGC, which is around 696 nm (inside the NIR imaging window, which ranges from 650 to 900 nm), is also consistent with the results found in the traditional solid‐state reaction.^20^, ^21^, ^22^ The PL of ZGC can be detected even after more than 5 h followed by an excitation for 5 min with an UV light source (254 nm; Figure 1D). Synthesis of zinc gallate based phosphors can usually only occur at temperatures at least 750 °C.^20^, ^21^, ^23^ The decrease in synthesis temperatures from those generally used implies that the utilization of the pores of MSNs as nanoreactors facilitates the formation of ZGC phosphor.^24^ We speculate that homogeneous distribution of these reactants in confined mesoporous nanostructures results in a higher reactivity than would otherwise be the case; such phenomena were observed previously.^25^, ^26^, ^27^, ^28^, ^29^

### 2.2 Morphology and Porous Structure of mZGC

**Figure 2:**
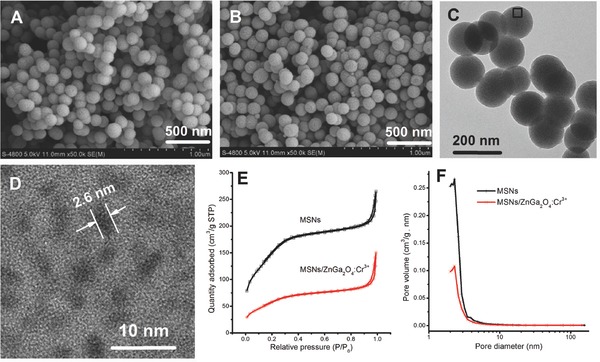
Morphologies and porous structure of MSNs and mZGC. FESEM images of A) MSNs and B) mZGC. C) TEM and D) HRTEM images of mZGC, E) N_2_ adsorption/desorption isotherms, and F) pore‐size distributions of MSNs and mZGC.

This substantial decrease in synthesis temperature is of vital importance to generate mZGC nanocomposites successfully since the nanochannels of MSNs start to collapse when annealed at temperatures higher than 600 °C.^30^ The field‐emission scanning electron microscopy (FESEM) images of the as‐synthesized MSNs (**Figure** **2**A) and the corresponding mZGC (Figure 2B) indicate that the MSNs survive calcination at 600 °C; no apparent morphological change could be observed. Furthermore, in the transmission electron microscopy (TEM) and high‐resolution‐TEM (HRTEM) images (Figure 2C,D), tiny dark spots (ZGC nanoparticles) appear homogeneously in the nanochannels of the MSNs. Since the mesoporous structure of the product is essential in our experiment, N_2_ adsorption/desorption was performed to study the influence of the synthesis process on the specific surface area and pore‐size distribution of the MSNs. Apparent mesoporous characteristics of plateau regions can be observed both for the MSN templates and for the as‐synthesized mZGC (Figure 2E). The specific surface area of the MSNs according to the Brunauer–Emmett–Teller (BET) method decreased from 554.2 to 214.6 m^2^ g^−1^, while the pore volume decreased from 0.3395 to 0.1562 cm^3^ g^−1^, which might arise from the formation of particles in the mesopores. The synthesis of mZGC from the MSNs did not decrease the overall average pore size of the carriers. In fact, a slight increase in average pore size from 2.450 to 2.912 nm was observed, as shown in Figure 2F, which may be explained by the ZGC existing as isolated, tiny particles in the nanochannels of the MSNs, and this is also evidenced by the HRTEM image. The mesoporous properties of the as‐synthesized mZGC were also verified by the sustained release of a widely used model cargo, ibuprofen (Figure S2, Supporting Information). Thus, our PL nanocarriers synergized both unique optical properties of ZGC and the cargo storage/release properties of MSNs.

### 2.3 Imaging Capability of mZGC Through Simulated Deep Tissue

**Figure 3:**
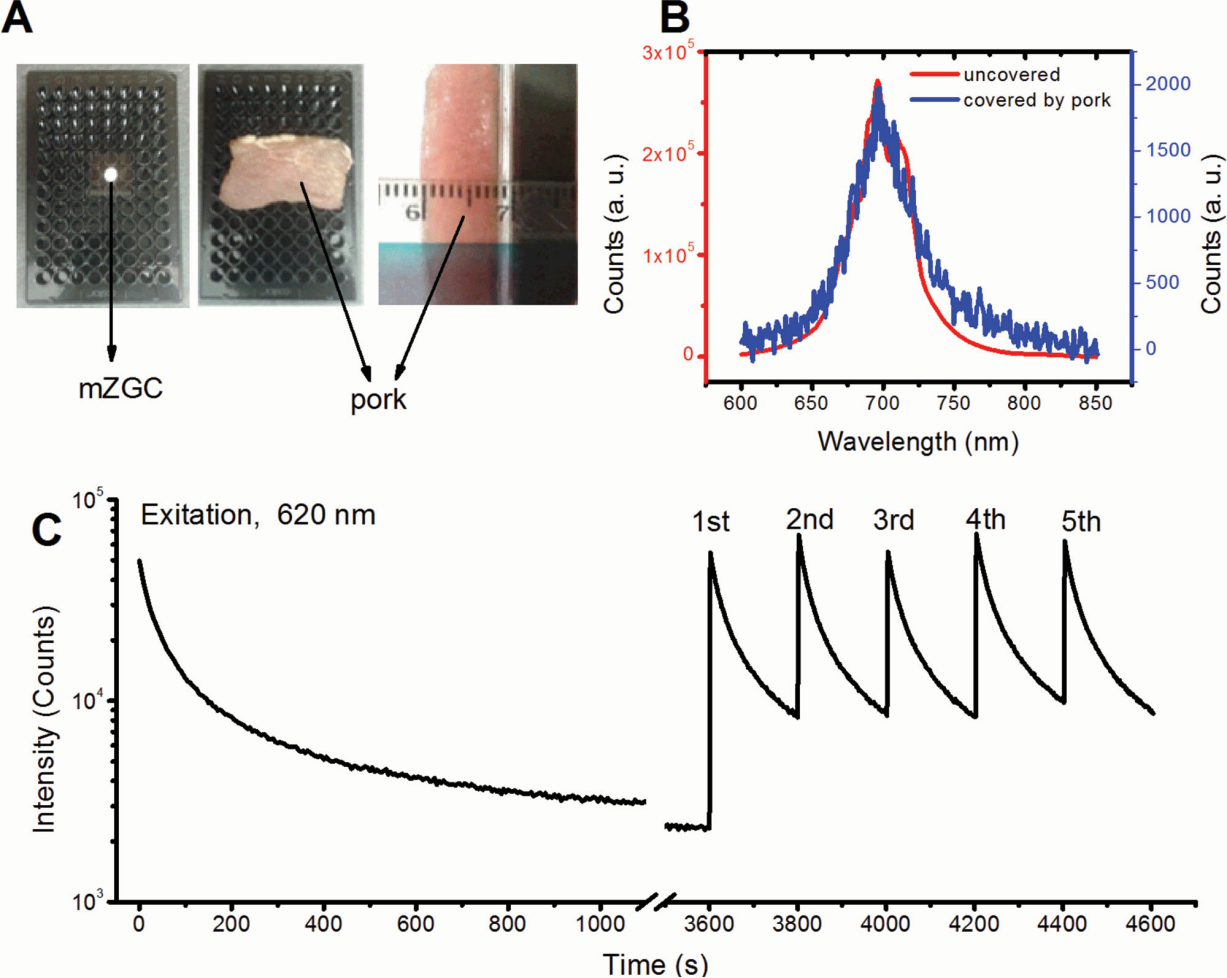
In situ simulated deep‐tissue charging properties of mZGC. A) Optical image of in situ excitation, B) PL spectra covered and uncovered by pork layer, C) in vitro charged and recharged decay curves of mZGC. All spectra were recorded with the ZGC under an 8 mm pork layer.

Most of the current PL phosphors can only be excited effectively under blue or even UV light which can hardly penetrate the deep tissue of animals.^1^, ^4^, ^7^ The PL excitation band from 600 to 650 nm is in the transmission window of biological tissue (600–1100 nm)^12^ and thus gives us the opportunity to recharge the energy‐exhausted mZGC in deep tissue in situ. Since there is no standard method to accurately evaluate the deep‐tissue‐imaging ability of PL phosphors, we propose a meat‐covering method for comparison according to our previous report on up‐conversion imaging (**Figure** **3**A).^31^ The as‐synthesized mZGC sample disk could be excited at 620 nm when covered by 8 mm of pork. After switching off the light used for excitation, the PL spectrum can be recorded by using a fluorospectrometer (FluoroMax‐3, HORIBA, USA) fitted with a photomultiplier tube (PMT) detector. A similar PL spectrum can be obtained to that of the uncovered sample, which means that the NIR PL of mZGC can efficiently penetrate pork tissue as thick as 8 mm, as shown in Figure 3B. The excited PL of mZGC could be reproduced consecutively in situ more than five times on any occasion, as shown in Figure 3C.

**Figure 4:**
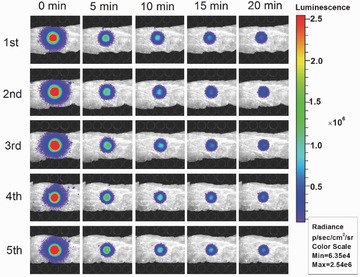
Recharged in vitro PL imaging of mZGC covered by an 8‐mm pork layer.

**Figure 5:**
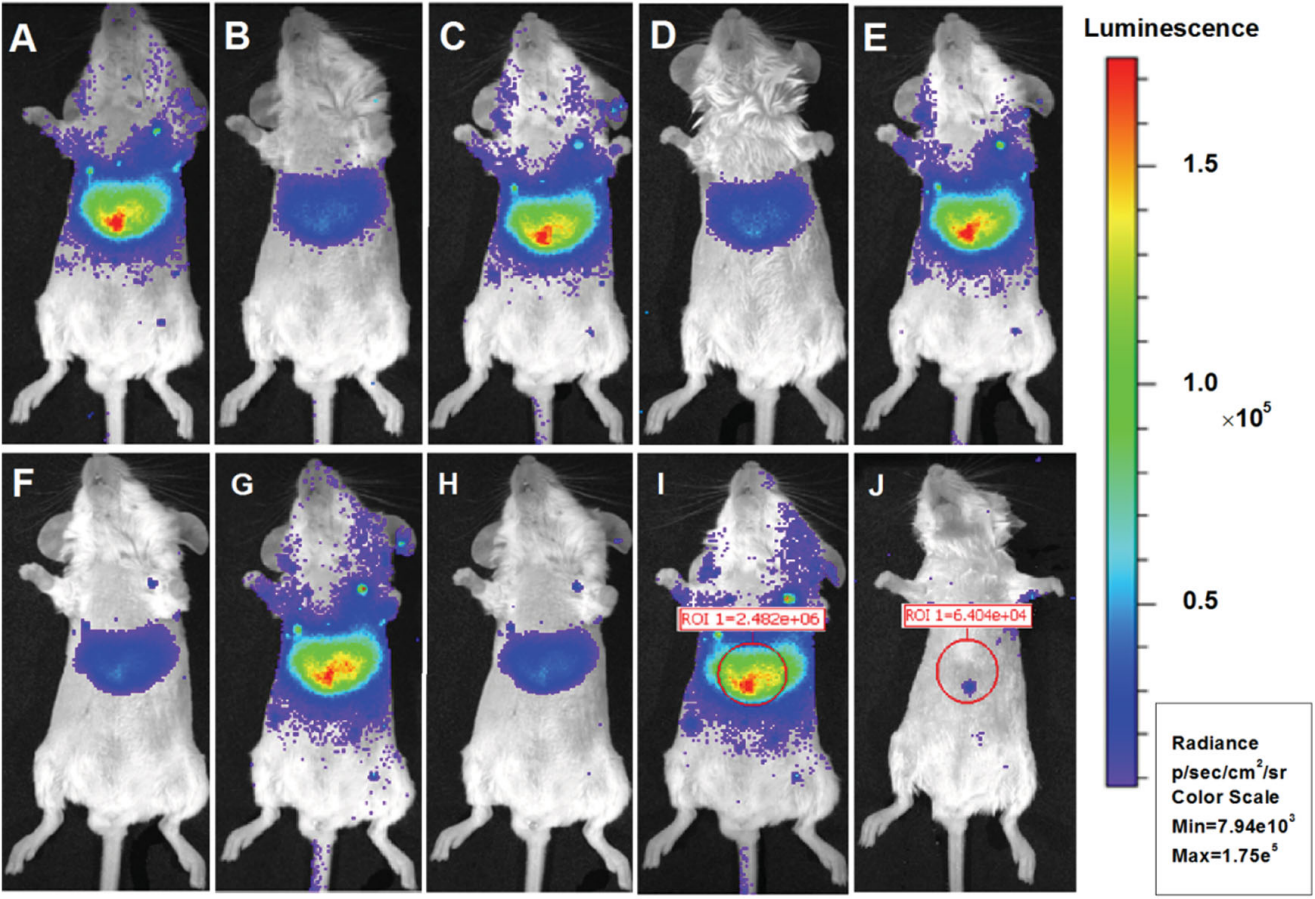
In vivo recharging of mZGC for PL imaging by using a white LED. A) First charging, B) 10 min after first charging, C, E, G, I) second to fifth recharging at time intervals of 10 min, D,F,H) 10 min after second, third, and forth recharging, J) background control imaging of a mouse without injection of mZGC.

**Figure 6:**
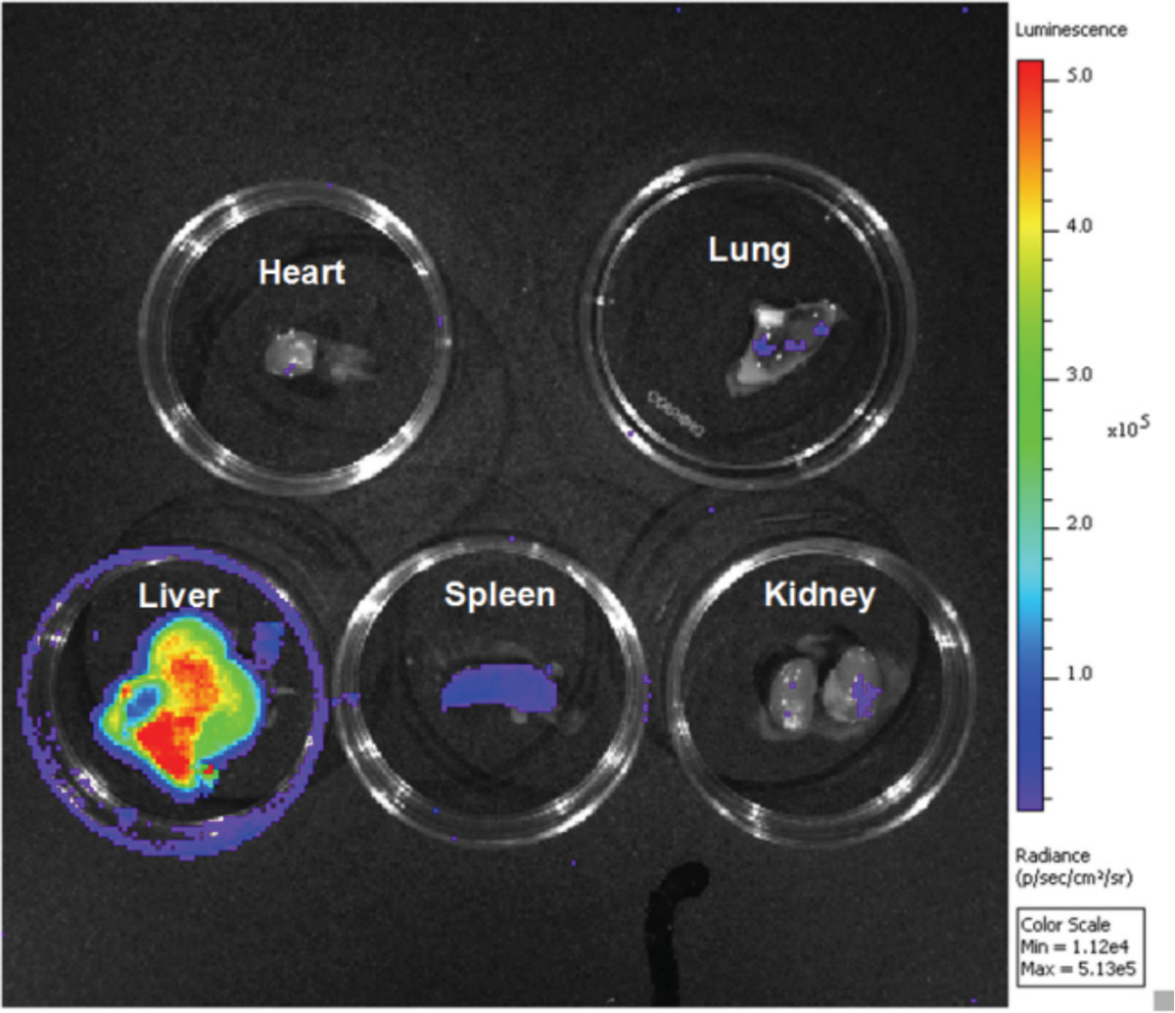
Biodistribution of mZGC, 2 h after tail‐vein injection.

Based on our observation that the PL of mZGC can be charged by red light under an 8 mm pork layer, for the first time we studied its applications in deep‐tissue imaging by using a white light‐emitting diode (LED) as the excitation light source (spectrum shown in Figure S4, Supporting Information). The rechargeable PL imaging of the mZGC sample was performed five times under a pork layer of 8 mm without any obvious signal weakening, which implies its potential for use in in vivo imaging applications (**Figure** **4**). For in vivo applications, good biocompatibility is anticipated since the NIR PL phosphor, ZGC, is mainly incorporated within the mesopores of the MSNs, which are well‐known to be biocompatible. No apparent cellular toxicity could be observed for mZGC under an exposure concentration as high as 50 mg L^−1^ (Figure S3, Supporting Information). To study the in vivo chargeable ability of mZGC, we injected the mZGC saline solution (200 μL, 5 mg mL^−1^) into a live mouse through the tail vein. After exposure to white LED light, a satisfactory PL imaging picture was obtained, as shown in **Figure** **5**. Moreover, after reperforming the in situ excitation, the PL of ZGC could be recharged again. No apparent decrease in PL signal was observed after five imaging/recharging cycles (Figure 5). A high signal‐to‐noise ratio of ≈40:1 was obtained by comparing the PL signal from liver area of mouse with and without injecting mZGC. The biodistribution of the mZGC was studied after euthanasia of the mouse (2 h after injection). Most of the PL signals emanate from the liver and spleen, which is consistent with the in vivo imaging results (**Figure** **6**). Given the in vivo chargeable PL attributes, the detection of the PL functionalized mesoporous carriers in vivo is not limited by the decay of the PL intensity. Thus, the as‐synthesized mZGC has great potential to become a new class of mesoporous nanocomposites with NIR PL properties.

## 3 Conclusions

We developed in vivo rechargeable near‐infrared‐emitting mesoporous SiO_2_/ZnGa_2_O_4_:Cr^3+^ persistently luminescent nanocomposites. By using the mesopores of MSNs as a reaction template, the synthesis temperature of the persistent phosphor, ZnGa_2_O4:Cr^3+^, was decreased from higher than 750 °C in a solid‐state reaction to only 600 °C. At this lower temperature, both the unique mesoporous attributes, and the uniform size and morphology of the MSNs were retained in the nanocomposites. For the first time, we confirmed that mZGC could be repeatedly charged in situ under a deep‐tissue layer of 8 mm. The deep‐tissue chargeable PL properties of mZGC also ensured its repeatable recharged PL imaging in a live mouse model. It is worth noting that this observation is the first direct evidence that persistent luminescence can be recharged in vivo for multiple times. This concept of utilizing mesoporous silica as nanoreactor to fabricate ZGC PL nanoparticles with uniform morphology and preserved porous nanostructure will be significant in directing the synthesis of mesoporous PL systems with diverse PL phosphors and paves a new way to the wide application of deep tissue rechargable ZGC in photonics and biophotonics.

## 4 Experimental Section

*Materials*: Tetraethoxysilane (TEOS), ethanol, diethanolamine (DEA), ammonium hydrate, cetyltrimethylammonium bromide (CTAB), Ga_2_O_3_, Zn(NO_3_)_2_·6H_2_O, Cr(NO_3_)_3_·9H_2_O, and concentrated nitric acid were all of analytical standards, purchased from Sigma‐Aldrich and were used as‐received. Ga(NO_3_)_3_ solution was prepared by dissolving Ga_2_O_3_ in 1:1 concentrated nitric acid followed by air drying at 105 °C to remove excess nitric acid, and then redissolving in deionized water.

*Synthesis of MSNs*: The MSN synthesis was modified from that in a previous report.^25^ Briefly, 7 mL of ethanol, 0.2 g of CTAB, and 50 μL of diethanolamine were dissolved in 25 mL of water under stirring at 60 °C for 30 min to prepare a transparent solution. Then, 2 mL of tetraethoxysilane were added rapidly. The reaction was finished after stirring for another 2 h. Mesoporous silica nanospheres (about 100‐nm diameter) were collected by centrifugation and calcination at 550 °C for 2 h to remove CTAB and possible organic residues.

*Synthesis of mZGC Nanocomposites*: A precursor solution was prepared by dissolving the corresponding nitrates in water/ethanol (1/1, v/v). The final concentration of Zn^2+^, Ga^3+^, and Cr^3+^ was controlled to be 0.5, 0.9985, and 0.0015 mol L^−1^, according to a stoichiometric ratio of Zn/Ga/Cr (1/1.997/0.003, molar ratio), respectively. 200 μL of the precursor solution was mixed with 200 mg mesoporous silica and the mixture was dried in a vacuum oven at 50 °C for 12 h. The samples were then put into a muffle furnace and the temperature was slowly increased by 5 °C min^−1^.

*In Vitro and In Vivo PL Imaging of mZGC*: In vitro imaging was performed by putting the powder (100 mg) sample into a black 96‐well plate covered with an 8‐mm layer of pork tissue. The PL signal from the covered mZGC was recorded after illumination by using an LED (5000 lumen) for 15 s. The in vivo imaging was conducted by injection of the mZGC dispersion in phosphate‐buffered saline (PBS; 5 mg mL^−1^) through the tail vein. The sample was stored in a dark box for 1 day before injection to ensure no preactivation occurred.

*Characterization*: X‐ray powder diffraction (XRD) measurements were performed on a diffractometer equipped with Cu Kα radiation (λ = 1.5418 Å) (Panalytical X'pert PRO, The Netherlands). The morphology of the samples was inspected by using field‐emission scanning electron microscopy (FESEM, HITACHI S‐4800, Japan) and transmission electron microscopy (TEM, HITACHI H‐7650, Japan) at accelerating voltages of 5 and 100 kv, respectively. High‐resolution TEM (HRTEM) images were recorded by using a JEM‐1200EX II transmission electron microscope. N_2_ adsorption/desorption isotherms were obtained on a full‐automatic physical and chemical adsorption apparatus (micromeritics, ASAP2020C, USA). Pore size distribution was calculated from the adsorption branch of N_2_ adsorption/desorption isotherm and the Brunauer–Emmett–Teller (BET) method. The BET specific surface areas were calculated using the data between 0.05 and 0.35 just before the capillary condensation. The total pore volumes were obtained by the *t*‐plot method. Total organic carbon analyzer was used to determine the exact loading level of ibuprofen on mZGC by using a CNS elemental analyzer (Elementar Portfolio, Vario MAX, Germany). The spectra and lifetimes of the samples were tested by using powdered samples. The photoluminescence spectra and time‐decay curves were measured by using a fluorospectrophotometer (FluoroMax‐3, HORIBA, USA). The absorbance was detected on an UV–vis spectrometer (Thermo, Evolution300, USA). The PL imaging of mZGC was conducted in a Xenogen IVIS imaging system.

## Supporting information

As a service to our authors and readers, this journal provides supporting information supplied by the authors. Such materials are peer reviewed and may be re‐organized for online delivery, but are not copy‐edited or typeset. Technical support issues arising from supporting information (other than missing files) should be addressed to the authors.

Supplementary
