# Supplementary material for: In Vivo Repeatedly Charging Near‐Infrared‐Emitting Mesoporous SiO2/ZnGa2O4:Cr3+ Persistent Luminescence Nanocomposites
Source: Adv Sci (Weinh). 2015 Feb 9;2(3):1500001. doi: 10.1002/advs.201500001 (PMC4941782; doi:10.1002/advs.201500001)
Supplement: Supplementary file 1 — Supplementary [file ADVS-2-0j-s001.pdf]

Copyright WILEY-VCH Verlag GmbH & Co. KGaA, 69469 Weinheim, Germany, 2015.

Supporting Information

for *Adv. Sci.,* DOI: 10.1002/advs.201500001

In Vivo Repeatedly Charging Near-Infrared-Emitting Mesoporous $SiO_2/ZnGa_2O_4:Cr^{3+}$ Persistent Luminescence Nanocomposites

*Zhanjun Li, Yuanwei Zhang, Xiang Wu, Xiaoqiong Wu, Rohit Maudgal, Hongwu Zhang, and Gang Han**

Supporting Information

In Vivo Repeatedly Charging Near-Infrared-Emitting Mesoporous $SiO_2/ZnGa_2O_4:Cr^{3+}$ Persistent Luminescence Nanocomposites

*Zhanjun Li, Yuanwei Zhang, Xiang Wu, Rohit Maudgal, Gang Han**

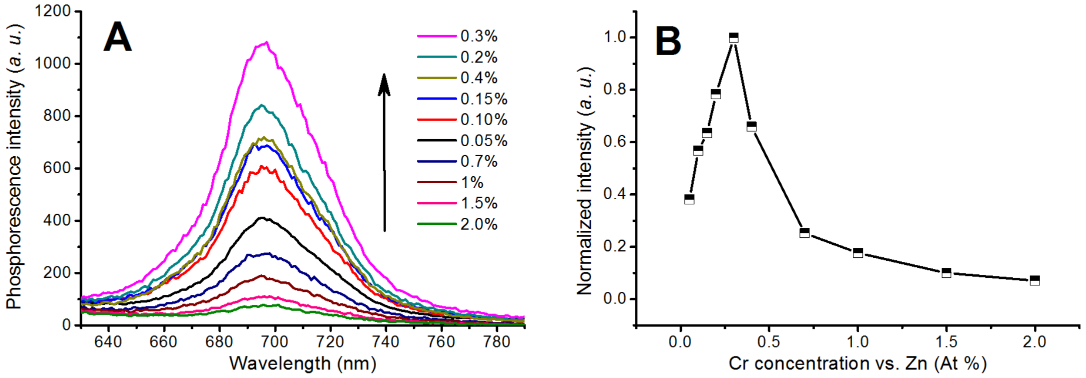

Figure S1. Optimization of Cr^{3+} doping concentration (vs. Zn) in mZGC.

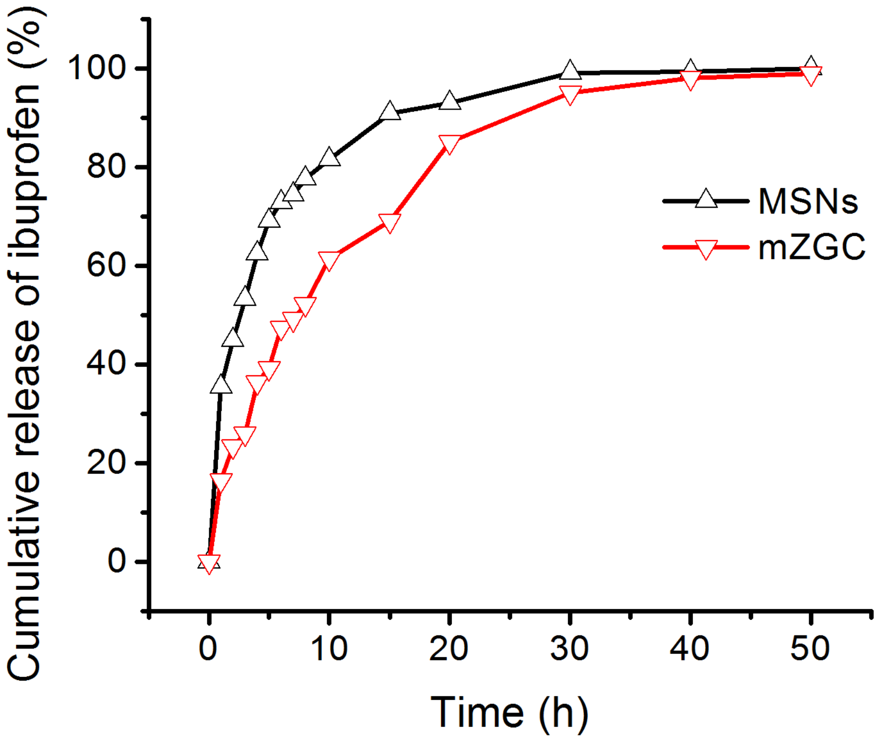

Figure S2. Ibuprofen storage/release properties of mZGC. The ibuprofen storage and release experiment was performed according to a previous report.[1] Briefly, 60 mg of the MSNs/$ZnGa_2O_4$:Cr^{3+} were immersed into 1 mL of 20 mg/L ibuprofen cyclohexane solution for 24 h. Excess ibuprofen cyclohexane solution was removed by centrifugation and decantation. The cyclohexane in the mesoporous silica was evaporated in a 50 °C air dryer for 1 h. The *in vitro* release of ibuprofen was performed by immersing 60 mg of the drug loaded mZGC in 60 mL of simulated body fluid at 37 °C. 100 μL of the mixed solution was taken to test the released amount of ibuprofen at fixed time intervals; this was centrifuged to determine the released ibuprofen in the supernatant by detecting the absorbance. The initial content of ibuprofen on MSNs and mZGC is 146.3 mg/g and 103.9 mg/g respectively, by elemental analysis.

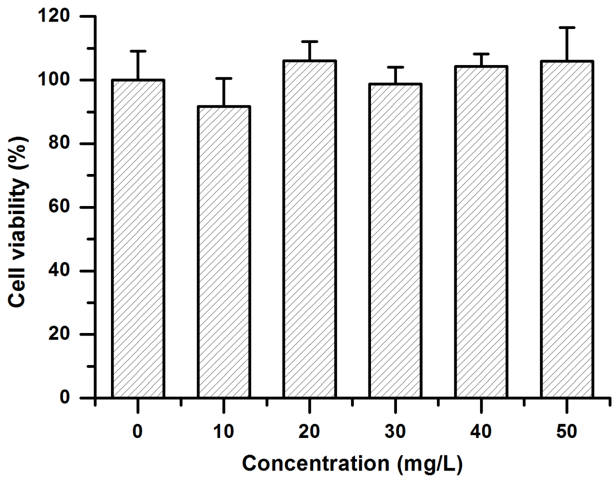

Figure S3. Cell toxicity of mZGC. The cytotoxicity was tested by using 3-(4,5-dimethyl-2-thiazolyl)-2,5diphenyl-2H-tetrazolium bromide (MTT, sigma, USA) assay by using human umbilical vein endothelial cells (HUVE cells, ATCC, CRL-1730).

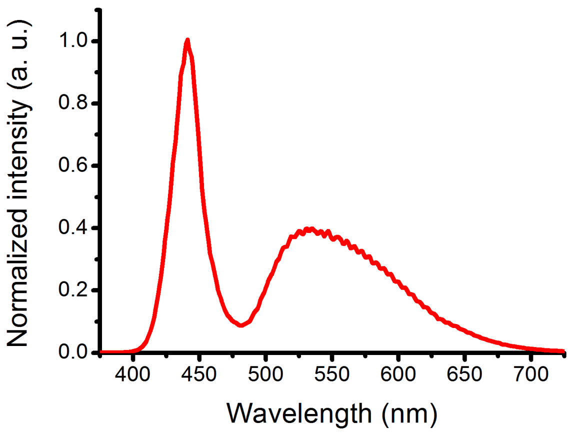

Figure S4. Spectrum of the LED torch used for our experiments. Wavelengths longer than 600 nm have better biotissue penetration ability than shorter ones, and result in the charging of mZGC *in vivo*.

Table S1 Test of the ZGC content in as-synthesized mZGC product.

MSNs	mZGC	Increased mass	ZGC content in mZGC (by weight)
400.0 mg	445.7 mg	45.7 mg	10.4%±0.4%
400.0 mg	448.1 mg	48.1 mg	
400.0 mg	445.4 mg	45.4 mg	

Table S2 Mass loss of mZGC after rinsed in PBS for 48 hours.

mZGC	Rinsed and dried mZGC	decreased mass	Mass loss by weight
100.0 mg	99.7 mg	-1.7 mg	0.2 %±1.5%
100.0 mg	101.4 mg	+1.4 mg	
100.0 mg	100.1 mg	+0.1 mg	

[1] P. P. Yang, S. S. Huang, D. Y. Kong, J. Lin, H. G. Fu, Inorg Chem 2007, 46, 3203.
